# Validation of a guideline-based decision support system for the diagnosis of primary headache disorders based on ICHD-3 beta

**DOI:** 10.1186/1129-2377-15-40

**Published:** 2014-06-16

**Authors:** Zhao Dong, Ziming Yin, Mianwang He, Xiaoyan Chen, Xudong Lv, Shengyuan Yu

**Affiliations:** 1 International Headache Center, Department of Neurology, Chinese PLA General Hospital, Fuxing Road 28, Haidian District, Beijing 100853, China; 2 College of Biomedical Engineering and Instrument Science, Zhejiang University, Hangzhou, Zhejiang 310008, China

**Keywords:** Headache, Computer-assisted diagnosis, Clinical decision support, ICHD-3 beta, SAGE

## Abstract

**Background:**

China may have the largest population of headache sufferers and therefore the most serious burden of disease worldwide. However, the rate of diagnosis for headache disorders is extremely low, possibly due to the relative complexity of headache subtypes and diagnostic criteria. The use of computerized clinical decision support systems (CDSS) seems to be a better choice to solve this problem.

**Methods:**

We developed a headache CDSS based on ICHD-3 beta and validated it in a prospective study that included 543 headache patients from the International Headache Center at the Chinese PLA General hospital, Beijing, China.

**Results:**

We found that the CDSS correctly recognized 159/160 (99.4%) of migraine without aura, 36/36 (100%) of migraine with aura, 20/21 (95.2%) of chronic migraine, and 37/59 (62.7%) of probable migraine. This system also correctly identified 157/180 (87.2%) of patients with tension-type headache (TTH), of which infrequent episodic TTH was diagnosed in 12/13 (92.3%), frequent episodic TTH was diagnosed in 99/101 (98.0%), chronic TTH in 18/20 (90.0%), and probable TTH in 28/46 (60.9%). The correct diagnostic rates of cluster headache and new daily persistent headache (NDPH) were 90.0% and 100%, respectively. In addition, the system recognized 32/32 (100%) of patients with medication overuse headache.

**Conclusions:**

With high diagnostic accuracy for most of the primary and some types of secondary headaches, this system can be expected to help general practitioners at primary hospitals improve diagnostic accuracy and thereby reduce the burden of headache in China.

## Background

Headache disorder is considered to be one of the most common reasons for medical consultation in both primary care units and neurological clinics [1,2]. The estimated 1-year prevalence of primary headache in China was 23.8%, including migraine (9.3%) and tension-type headache (TTH) (10.8%). With a population of over 1.3 billion, it is estimated that China may have the largest headache population worldwide. Therefore, the total burden of primary headache disorders in China is very serious, accounting for CNY 672.7 billion (USD 96.9 billion) or 2.24% of GDP [3]. However, the correct rate of diagnosis for headache disorders is extremely low. A recent population-based door-to-door survey conducted in China reported rates of 13.8% for migraine and 2.6% for TTH [4]. A number of nonstandard headache diagnoses such as “vascular headache” and “nervous headache” are still widely applied in clinical practice throughout China [4]. These nonstandard diagnoses may result in inappropriate treatment measures such as analgesic drug abuse, unnecessary auxiliary examinations such as magnetic resonance imaging (MRI), and repeated consultations, which may aggravate the burden of headache disorders.

The International Classification of Headache Disorders, Second Edition (ICHD-II) [5] is available for the diagnosis of headache disorders worldwide. A revised 3rd edition (ICHD-3, beta version) was also published last year [6]. However, the relative complexity of headache subtypes and diagnostic criteria may confuse many general practitioners and even neurological physicians in primary and secondary care who are not familiar with the ICHD criteria.

The clinical decision support system (CDSS) is interactive decision support system (DSS) computer software, which is designed to assist clinicians and other health professionals with decision making tasks. CDSS has been effective in improving outcomes at some healthcare institutions and practice sites by making medical knowledge readily available to users [7]. A substantial body of evidence exists to suggest that DSS can be extremely effective for improving clinical practice, especially the guideline-based CDSS [8-11]. Thus, the use of CDSS, as an assistant decision-making tool which can help clinicians to solve complex medical problems, seems to be a good choice in the management of headache.

At present, there are few reports utilizing CDSS technology to diagnose headache disorders. Maizels et al. [12] has developed an on-line Computerized Headache Assessment Tool (CHAT). However, the tool was designed for patients not for clinicians. Mainardi et al. [13] also developed a computerized program to assist general practitioners in the diagnosis of the principal forms of primary headaches. However, it is based on the ICHD-II criteria, rather than the latest criteria. Based on ICHD-II criteria, Sarchielli et al. [14] used the Primary Headaches Analyzer 1.0 INT software, to diagnose primary chronic headaches but the headache types covered by the software are limited. To the best of our knowledge, although some methods or systems have been developed, none is based on ICHD-3 and reflects the latest research on headache disorders. In addition, the knowledgebase of most systems is rule-based and lacks a complete computerized clinical guideline model, as well as the integration of electronic medical records. Therefore, it is difficult for these systems to be widely used in the actual clinical setting.

Our previous study completed the preliminary CDSS and validated it retrospectively with the clinical information from outpatients with a confirmed diagnosis of headache [15]. Although the CDSS indicated superior diagnostic accuracy for some types of headaches such as migraine, TTH and cluster headache, the system did not include other subtypes of headaches such as migraine with aura, and new daily persistent headache (NDPH). In addition, it was not based on the new ICHD criteria. Therefore, the purpose of the current study was firstly to revise the CDSS on the basis of ICHD-3 beta and secondly, to prospectively apply this CDSS to the diagnosis of outpatients and evaluate its sensitivity and specificity in our headache clinic. Based on this, we aimed to develop a CDSS to help community doctors, general practitioners and inexperienced physicians to simplify clinical diagnostic procedures and increase diagnostic accuracy, in order to improve the level of diagnosis and lift the burden of headache disorders in China.

## Methods

### Ethics

The study protocol was approved by the ethics committee of the Chinese PLA General Hospital, Beijing, China.

### Knowledgebase development

**Figure 1:**
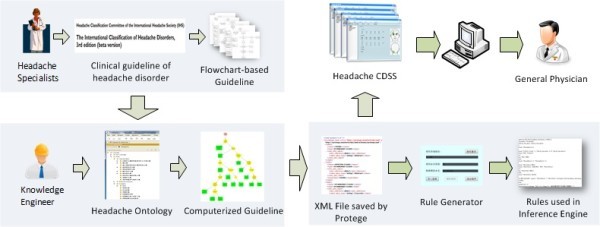
The development process of headache knowledge.

Building a knowledgebase is an essential step in the development of CDSS. A knowledgebase consists of a series of rules understood and executed by a computer. In our research, these rules were derived from a computerized clinical guideline model. Because a computerized clinical guideline model is totally different from text-based guidelines in format, it is difficult for clinical specialists to translate one format into another. Meanwhile, due to a lack of the necessary medical knowledge, this work is unsuitable to be completed by knowledge engineers even though they are familiar with computerized clinical guidelines. Therefore, the construction of a medical knowledgebase should rely on the joint efforts of clinical specialists and knowledge engineers. Based on this, we proposed a method of constructing a computerized clinical guideline model and medical knowledgebase, as shown in Figure 1.

First of all, headache specialists translated diagnostic knowledge in text-based guideline (ICHD) into a thought process of headache diagnosis. As the ICHD does not include the overall thought process or inference procedure of headache diagnosis, clinical specialists have to summarize it according to their own clinical experience, and then express it in the form of a flowchart. We selected a flowchart as the form of expression because it could be easily understood by both headache specialists and knowledge engineers.

Then, knowledge engineers established the computerized clinical guideline representation model according to these flow charts by referencing the modeling method of SAGE (standards-based sharable active guideline environment) [16]. First, we defined the domain ontology on headache diagnosis. Then, some necessary clinical data modules were set up in the Data Module class of SAGE. Thirdly, we summarized the logical comparison expression in headache diagnosis and defined them in the Expression class of SAGE. The activity graph of SAGE was utilized to express the inference procedure of headache diagnosis. To date, the computerized clinical guideline model has been developed.

Finally, the computerized clinical guideline representation module of headache diagnosis cannot be directly executed by computer although it has been represented. It must be translated into rules that can be executed in the inference engine of CDSS. A rule consists of two parts: LHS (left hand side) is the conditions of a rule and RHS (right hand side) is the reasoning results. Three elements, Decision, Action and Context, are the three indispensable parts of the computerized clinical guideline representation module. Therefore, we mapped the three elements to the two parts of the rule in order to build a knowledge base. The mapping method is shown in Table 1. Triggering events in Contexts and conditions in Decisions were mapped to LHS to choose scenarios and make decisions and Recommendations in action elements via the RHS while the rules were fired. We developed a computer program called the rule generator to complete this mapping work automatically.

**Table 1:** Map between guideline element and rule

**Rule Element**	**LHS**	**RHS**
Context	Triggering Event	
Decision	Conditions	
Action		Recommendations

LHS, left hand side; RHS, right hand side.

### Validations

We applied the CDSS in a perspective study including 543 headache patients at the International Headache Center of the Chinese PLA General hospital from July 2013 to November 2013. Clinical information for headache disorders such as location, duration, attack frequency, severity, accompanying symptoms, and aura were also input into the CDSS by doctors who were unfamiliar with and blind to the diagnostic criteria of headaches. The CDSS then made a computerized diagnosis. Two different qualified and experienced specialists in headache neurology reviewed this information and made the final diagnosis, which was regarded as the gold standard. Then, for each patient, the specialist’s diagnosis and CDSS’s diagnosis were compared. For the patient who experienced more than one type of headache, we focused solely on the most troubling one.

### Statistics

SPSS for Windows (Version 18.0) software was used for statistical analyses. Sensitivity, specificity and positive (PPV) and negative predictive values (NPV) were calculated for the CDSS diagnoses of headache disorders including migraine with (MO) or without aura (MA), probable migraine (PM), chronic migraine (CM), frequent episodic TTH (fETTH), infrequent episodic TTH (iETTH), probable TTH (PTTH), chronic TTH (CTTH), cluster headache (CH), new daily persistent headache (NDPH), medication overuse headache (MOH), and unclassified headaches against the gold standard. Cohen’s kappa (κ) was calculated for the agreement between diagnoses. Guidelines suggest that values of κ above 75% indicate excellent agreement, values between 75 and 40% indicate good to fair agreement and those below 40% show poor agreement [17]. A 5% level of significance and 95% confidence intervals (CI) were utilized.

## Results

The CDSS correctly recognized 159/160 (99.4%) of patients with MO, 36/36 (100%) of patients with MA, 20/21 (95.2%) of patients with CM, and 37/59 (62.7%) of patients with PM (Table 2). This system also correctly identified 157/180 (87.2%) patients with TTH, of which iETTH was diagnosed in 12/13 (92.3%), frequent episodic TTH was diagnosed in 99/101 (98.0%), CTTH in 18/20 (90.0%), and probable TTH in 28/46 (60.9%). The correct recognition rate of cluster headache and NDPH were 90.0% and 100%, respectively. In addition, the system recognized 32/32 (100%) patients with MOH.

**Table 2:** Agreement between clinical decision support system and headache specialist diagnoses

	**Specialist**													
**CDSS**	**Migraine**				**TTH**				**PM + PTTH**	**CH**	**NDPH**	**MOH**	**Others**	**Total**
	**MO**	**MA**	**CM**	**PM**	**iETTH**	**fETTH**	**CTTH**	**PTTH**						
Migraine														
MO	159			7		1		4	(4)					167
MA		36												36
CM			20				2							22
PM				37	1	1				2			2	43
TTH														
iETTH					12					1				13
fETTH						99							2 (※)	101
CTTH			1				18						2	21
PTTH								28					10 (#)	38
CH				2						27				29
NDPH											14			14
MOH												32		32
Others	1			13				14	(13)				12 (*)	27
**Total**	160	36	21	59	13	101	20	46		30	14	32	28	543

*MO*: migraine without aura; *MA*: Migraine with aura; *CM*: chronic migraine; *PM*: probable migraine; *TTH*: tension-type headache; *iETTH*: infrequent episodic tension-type headache; *fETTH*: frequent episodic tension-type headache; *CTTH*: chronic tension-type headache; *PTTH*: probable tension-type headache; *CH*: cluster headache; *PCH*: probable cluster headache; *NDPH*: new daily-persistent headache; *MOH*: medication overuse headache ※: 1 patient satisfied 7.6 Headache attributed to epileptic seizure and 1 patient for 14. Other headache disorders; #: 6 patients satisfied 13. Painful cranial neuropathies and other facial pain, 2 patients for 14. Other headache disorders, 1 patient for 4.5 Cold-stimulus headache and 1 patient for 11.2.1 Cervicogenic headache; *: 4 patients satisfied 13. Painful cranial neuropathies and other facial pains, 1 patient for 4.3 Primary headache associated with sexual activity, and 5 patients for 14. Other headache disorders.

The sensitivity, specificity, PPV and NPV of the CDSS for headache disorders were calculated and are presented in Table 3. These results demonstrated that the CDSS was accurate and reliable in diagnosing MO (sensitivity 99.38%, specificity 97.91%, kappa = 0.9606), MA (sensitivity 1, specificity 1, kappa = 1), CM (sensitivity 95.24%, specificity 99.62%, kappa = 0.9274), iETTH (sensitivity 92.31%, specificity 99.81%, kappa = 0.9212), fETTH (sensitivity 98.02%, specificity 99.55%, kappa = 0.9757), CTTH (sensitivity 90%, specificity 99.43%, kappa = 0.8733) and CH (sensitivity 90%, specificity 99.61%, kappa = 0.91). However, sensitivity was relatively low for PM (62.71%) and PTTH (60.87%), despite a very high specificity (98.76% for PM and 97.99% for PTTH). The value of κ indicated fair agreement for PM (0.6978) and PTTH (0.639). With regard to MOH and NDPH, CDSS displayed perfect sensitivity and specificity (1 for both headaches).

**Table 3:** Statistical indices of clinical decision support system diagnostic performance

	**Migraine**				**TTH**				**CH**	**MOH**	**NDPH**
	**MO**	**MA**	**CM**	**PM**	**iETTH**	**fETTH**	**CTTH**	**PTTH**			
Sensitivity (%)	99.38	100	95.24	62.71	92.31	98.02	90	60.87	90	100	100
(95% CI)	96.55- 99.89		77.33-99.15	49.95-73.92	66.69- 98.63	93.07-99.46	69.9-97.21	46.46-73.61	74.38-96.54		
Specificity (%)	97.91	100	99.62	98.76	99.81	99.55	99.43	97.99	99.61	100	100
(95% CI)	95.93- 98.94		98.61-99.89	97.32-99.43	98.94-99.97	98.37- 99.88	98.33-99.8	96.34-98.9	98.59-99.89		
Coincidence (%)	98.34	100	99.45	94.84	99.63	99.26	99.08	94.84	99.08	100	100
(95% CI)	96.88-99.13		98.39- 99.81	92.65-96.41	98.67-99.9	98.12-99.71	97.86-99.61	92.65-96.41	97.86-99.61		
False negative rate	0.01	0	0.05	0.37	0.08	0.02	0.10	0.39	0.10	0	0
False positive rate	0.02	0	0.01	0.02	0.01	0.01	0.01	0.02	0.01	0	0
Youden index	0.97	1	0.94	0.61	0.92	0.98	0.89	0.59	0.90	1	1
PPV (%)	95.21	100	90.91	86.05	92.31	98.02	85.71	73.68	93.1	100	100
(95% CI)	90.83-97.55		72.18-97.47	72.74- 93.44	66.69-98.63	93.07-99.46	65.36-95.02	57.99-85.03	78.04-98.09		
NPV (%)	99.73	100	99.81	95.6	99.81	99.55	99.62	96.44	99.42	100	100
(95% CI)	98.51-99.95		98.92- 99.97	93.43-97.08	98.94-99.97	98.37-99.88	98.61-99.89	94.44-97.73	98.3-99.8		
κ	0.9606	1	0.9274	0.6978	0.9212	0.9757	0.8733	0.639	0.910	1	1
(95% CI)	0.8766-1.045		0.8433- 1.011	0.615 - 0.7807	0.8371 - 1.005	0.8916 - 1.06	0.789-0.957	0.555-0.723	0.826-0.995		

*MO*: migraine without aura; *MA*: Migraine with aura; *CM*: chronic migraine; *PM*: probable migraine; *TTH*: tension-type headache; *iETTH*: infrequent episodic tension-type headache; *fETTH*: Frequent episodic tension-type headache; *CTTH*: chronic tension-type headache; *PTTH*: probable tension-type headache; *CH*: cluster headache; *MOH*: medication overuse headache; *CI*: confidence interval; *PPV*: positive predictive value; *NPV*: negative predictive value; *κ*: Cohen’s kappa.

## Discussion

In our study, CDSS for headache disorders has demonstrated a high degree of accuracy in recognizing MO, MA, CM, iETTH, fETTH, CTTH and cluster headache, and a fair degree of accuracy in distinguishing PM and PTTH. It has also shown perfect recognition of NDPH and MOH. Compared with previous computerized diagnostic tools, the CDSS performed in the current study has several advantages. Firstly, it is based on ICHD-3 beta, the newly published diagnostic tool. For example, based on the CDSS, TTH-like characteristic features are no longer needed for the diagnosis of NDPH. Also, for the diagnosis of MOH, ICHD-3 beta criteria removes the item “headache developed or markedly worsened during medication overuse” making the diagnosis of MOH more relaxed. Secondly, we standardized the computerized guideline model by referencing the modeling method of SAGE, and comparing it with the other headache CDSS. Its data model is based on HL7 V3 RIM [18] and the medical terminology is based on SNOMET CT [19]. The application of these standards allows the headache CDSS to integrate into electronic medical systems more easily, which will ensure greater convenience for clinicians. Thirdly, the proposed approach for knowledgebase development for CDSS provides a feasible method of collaboration between clinical specialists and knowledge engineers. To some extent, it provides a new methodology to solve the bottleneck problems of knowledge acquisition in CDSS fields. Last but not least, with the development of modern medicine, the content of clinical guidelines needs to be continually updated. Therefore, the method of constructing a knowledgebase must be easily maintained and updated. Compared with traditional CDSS (clinical knowledge and inferencing or control knowledge are mixed in the same representation), the rules in our system are separate from the program of headache CDSS, and thus updating of the knowledge base is convenient for system development engineers, they need only modify the rules in the knowledgebase instead of revising and compiling the whole program.

Our previous clinic-based study indicated that of all the headache patients, 78.4% were diagnosed as primary headaches, including migraine, TTH, and cluster headache [20]. Compared with secondary headaches, the diagnostic assessment of primary headaches depends mostly on clinical features such as duration, location, and accompanying symptoms, and thus lacks biochemical and neuroradiological markers. Therefore, we mainly focused CDSS on the primary headache diagnosis. Although secondary headaches accounted for 12.9% of all headaches, MOH was the most common subtype (≈7.4%) [20]. Therefore, we also paid more attention to this type of headache. With regard to other secondary headaches, we performed “red flags”, such as “headache occurred in patients aged more than 50 years old”, “headache which is the most painful ever experienced”, “headache with positive neurologic signs”, “sudden onset headache”, to exclude factors such as tumor, stroke, and infection prior to the diagnosis of primary headaches.

In this study, the reliability of a computerized clinical guideline method was verified in the form of diagnostic accuracy of the headache decision support system. The evaluation of this system has been performed by a comparison of the diagnostic accuracy of the system versus the gold standard identified by headache specialists. For migraine subtypes, we found high sensitivity, specificity, PPV and NPV for MO, MA and CM, with κ = 0.9606, 1, and 0.9274, indicating excellent agreement. However, for PM, κ = 0.6978 indicated only fair to good agreement. With regard to TTH subtypes, excellent agreement was obvious for iETTH (κ = 0.9212), fETTH (κ = 0.9757) and CTTH (κ = 0.8733). However, κ = 0.639 revealed an unsatisfactory diagnostic accordance rate for PTTH. There may be two reasons for the discrepancy. Firstly, the clinical presentations of migraine and TTH can often overlap in individual patients. Specialists can diagnose these patients as PM and/or PTTH based on their clinical experience. However, these ill-defined boundaries for headache features may confuse CDSS and thus inconsistent output may arise. Secondly, some patients experience simultaneous migraine and TTH, doctors who applied CDSS may not pay enough attention to “the troubled headache” the patient was experiencing at presentation. Therefore, it was difficult for the computer to identify mixed headache profiles and which headache was the most troubling headache according to ICHD-II criteria, leading to inconsistent diagnostic results.

In our study, κ for cluster headache was 0.91. The most common reason for the difference between CDSS and the specialist may be due to overlapping of both cluster headache and migraine [21-23]. Although the two disorders are distinct clinical entities, patients sometimes present with clinical symptoms having characteristics of both headache types. However, neither disorder fully complies with ICHD criteria. For example, in our series a patient has recurrent onset of migraine headache except for a headache duration of less than 4 hours. Also, the headache attacks in this patient have a seasonal periodicity but lack cranial autonomic symptoms (CAS) such as lacrimation or rhinorrhea. So, it appeared that probable migraine and probable cluster headache were both present. Another patient with cyclic and unmigrainous headache was diagnosed with TTH by CDSS based on ICHD criteria. On the other hand, CAS, which are distinguishing features of trigeminal autonomic cephalalgias, can also occur in patients with migraine. Although the CAS for migraine patients tended to be bilateral, strictly unilateral symptoms may confuse the CDSS, leading to misdiagnosis.

In addition to providing an auxiliary diagnosis, the CDSS also has an educational function by helping general practitioners familiar with ICHD criteria when clinical information for the diagnosis of headache was input and computerized diagnosis was output by the system. The system also has a diagnostic-conformation function: the general practitioners could press the “confirm” button when they agreed with the diagnostic recommendation of the system, if not, the physician could write his own diagnosis. Additionally, this system can provide treatment information based on international clinical guidelines. We have also completed a web-based CDSS for headache disorders and look forward to applying and verifying the system in a multicenter study. Briefly, our final aim is to implement this CDSS in primary care units and community hospitals, to improve the level of diagnosis and treatment for headache and finally reduce the burden of headache in China. Although the CDSS demonstrated satisfying diagnostic accuracy for headache disorders, it needs to be emphasized that this computer assisted diagnosis system does not take the place of the neurologist’s experience.

## Conclusion

The study is the first step in developing a computer-based diagnostic tool. The first testing phase in the headache clinic delivered promising results, which revealed a high degree of accuracy in recognizing MO, MA, CM, iETTH, fETTH, CTTH, cluster headache, NDPH, and MOH, and a fair degree of accuracy in distinguishing PM and PTTH. In the next step, the tool should be tested to determine its validity in primary care. The final aim of the study is to disseminate this CDSS and improve the level of diagnosis and treatment for headache in primary care units and community hospitals and thereby reduce the burden of headache in China.

## Consent

Written informed consent was obtained from the patient for the publication of this report and any accompanying images.

## Competing interests

All authors declare there are no financial competing interests (political, personal, religious, ideological, academic, intellectual, commercial or any other) in relation to this manuscript.

## Authors’ contributions

ZD, ZY, MH, XC and XL carried out the studies. ZD and ZY participated in the design of the study and drafted the manuscript. ZD and MH performed the statistical analysis. Professor SY, the PI of this study, conceived of the study and participated in its design and helped to draft the manuscript. All authors read and approved the final manuscript.
